# Evaluation of Genotoxic Pressure along the Sava River

**DOI:** 10.1371/journal.pone.0162450

**Published:** 2016-09-15

**Authors:** Stoimir Kolarević, Mustafa Aborgiba, Margareta Kračun-Kolarević, Jovana Kostić, Predrag Simonović, Vladica Simić, Aleksandra Milošković, Georg Reischer, Andreas Farnleitner, Zoran Gačić, Radmila Milačič, Tea Zuliani, Janja Vidmar, Marija Pergal, Marina Piria, Momir Paunović, Branka Vuković-Gačić

**Affiliations:** 1 University of Belgrade, Faculty of Biology, Chair of Microbiology, Center for Genotoxicology and Ecogenotoxicology, Belgrade, Serbia; 2 University of Belgrade, Institute for Biological Research ¨Siniša Stanković¨, Belgrade, Serbia; 3 University of Belgrade, Institute for Multidisciplinary Research, Belgrade, Serbia; 4 University of Belgrade, Faculty of Biology, Institute of Zoology, Belgrade, Serbia; 5 University of Kragujevac, Faculty of Science, Kragujevac, Serbia; 6 TU Wien, Institute for Chemical Engineering, Research Group Environmental Microbiology and Molecular Ecology, Interuniversity Cooperation Centre Water & Health, Vienna, Austria; 7 Department of Environmental Sciences, Jožef Stefan Institute, Ljubljana, Slovenia; 8 University of Belgrade, Institute of Chemistry, Technology and Metallurgy, Belgrade, Serbia; 9 University of Zagreb, Faculty of Agriculture, Zagreb, Croatia; Jinling Institute of Technology, CHINA

## Abstract

In this study we have performed a comprehensive genotoxicological survey along the 900 rkm of the Sava River. In total, 12 sites were chosen in compliance with the goals of GLOBAQUA project dealing with the effects of multiple stressors on biodiversity and functioning of aquatic ecosystems. The genotoxic potential was assessed using a complex battery of bioassays performed in prokaryotes and aquatic eukaryotes (freshwater fish). Battery comprised evaluation of mutagenicity by SOS/*umuC* test in *Salmonella typhimurium* TA1535/pSK1002. The level of DNA damage as a biomarker of exposure (comet assay) and biomarker of effect (micronucleus assay) and the level of oxidative stress as well (Fpg—modified comet assay) was studied in blood cells of bleak and spirlin (*Alburnus alburnus/Alburnoides bipunctatus* respectively). Result indicated differential sensitivity of applied bioassays in detection of genotoxic pressure. The standard and Fpg—modified comet assay showed higher potential in differentiation of the sites based on genotoxic potential in comparison with micronucleus assay and SOS/*umuC* test. Our data represent snapshot of the current status of the river which indicates the presence of genotoxic potential along the river which can be traced to the deterioration of quality of the Sava River by communal and industrial wastewaters. The major highlight of the study is that we have provided complex set of data obtained from a single source (homogeneity of analyses for all samples).

## Introduction

The Sava River is the major drainage basin of Southeastern Europe and the largest tributary to the Danube River. The river is 945 km long with catchment area of 97,713 km^2^ extending over Slovenia, Montenegro, Bosnia and Herzegovina, Croatia and Serbia [1]. The upper reaches of the Sava River basin are affected by hydromorphological pressures; the middle reaches by agricultural activities and eutrophication, and the lower reaches by industrial and urban pollution [2].

The population in the basin is about 8.2 million (46% of the total population of the four countries that share the basin) and the major issue is represented by high quantities of wastewaters which are in some sections processed and in the others unprocessed at all, due to different legislative and economic standards of the countries which share this international river. Preliminary data show that only 5.5% of the water bodies within the basin are characterized by high, 44.8% by good, 39.9% by moderate and 9.3% by poor ecological status [3].

But do we need to wait until the pollution pressure leaves the mark at the level of the ecosystem? Presence of various stressors in the environment (primarily organic and inorganic pollutants) can influence the integrity of DNA molecules in aquatic organisms which can have consequences on individual and population levels [4]. In the study of Ščančar et al. [5], moderate pollution by substances with nothworty genotoxic potential such as polycyclic aromatic hydrocarbons, pesticides and metalls (Hg, Cr, Ni) was detected at several sites along the Sava River. Therefore, ecogenotoxicological bioassays could become essential as early warning systems for the possible deterioration of the ecosystem health.

The study of Kittinger et al. [6] raised the question whether the assessment of ecological status by Directive 2000/60/EC covers all the needs for a comprehensive classification of water quality. In this study authors indicated importance of bacterial indicators of faecal pollution and subsequent consequence in means of the mutagenic and toxic pollution at contaminated sites. In our previous research [1] we have performed preliminary screening of the faecal pollution along the Sava River. The results indicated serious deterioration of water quality at some of investigated sites, emphasizing the importance for further assessment of genotoxic potential there.

The study of Smital and Ahel [7] provides comprehensive overview of ecogenotoxicological studies performed on the Sava River so far. Majority of these studies are focused on the section stretching from the Slovenian-Croatian border up to the confluence of the Una River [8–11]. This section is especially interesting as one of the largest urban settlements (Zagreb—capitol of Croatia) is situated here. Within the mentioned studies authors employed bioassays on freshwater fish (comet assay, micronucleus test) and prokaryotic bioassays (i.e. Ames test on *Salmonella typhimurium*). In our previous research we were focused on the lower stretch of the Sava River which comprises the urban area of Belgrade city [12–14]. Hereby the research was performed on freshwater mussels and freshwater fish (comet assay). Although the results of these studies represent a valuable and solid data set, these two sections of the river are only two pieces of the puzzle and a more comprehensive study is needed to provide the complete picture of genotoxic potential on the whole river level. Moreover, Smital and Ahel [7] emphasized that many of the results obtained in the middle section of the river may be outdated because in the meanwhile the city of Zagreb has implemented wastewater treatment facility which probably changes the previously recorded situation.

Having that in mind, the primary goal of our study was to identify the hotspots of pollution along the Sava River. Hotspots of faecal pollution were identified by bacterial indicators (*Escherichia coli* numbers), while hotspots related to industry were identified by assessing the concentrations of metals in tissues of spirlin *Alburnoides bipunctatus* (Bloch 1782) and/or bleak *Alburnus alburnus* (Linnaeus 1758), depending on the stretch of the Sava River. The second goal was to evaluate the genotoxic potential along the Sava River using a complex battery of bioassays performed in prokaryotes and aquatic eukaryotes (freshwater fish). Battery comprised evaluation of mutagenicity by SOS/*umuC* test in *Salmonella typhimurium* TA1535/pSK1002. Regarding the eukaryotes, the level of DNA damage as a biomarker of exposure (comet assay) and biomarker of effect (micronucleus assay) and the level of oxidative stress as well (Fpg—modified comet assay) were studied in blood cells of spirlin and bleak. Finally, we wanted to investigate whether the variation in genotoxic potential along the river can be linked to hotspots of faecal and industrial pollution. The study was carried out along 900 rkm of the Sava River including also upper and middle sections which were not studied before. The sites were chosen in compliance with the goals of GLOBAQUA project dealing with the effects of multiple stressors on biodiversity and functioning of aquatic ecosystems. The sites are included in national routine monitoring program of Slovenia, Croatia and Serbia.

## Materials and Methods

### Sampling area

**Fig 1:**
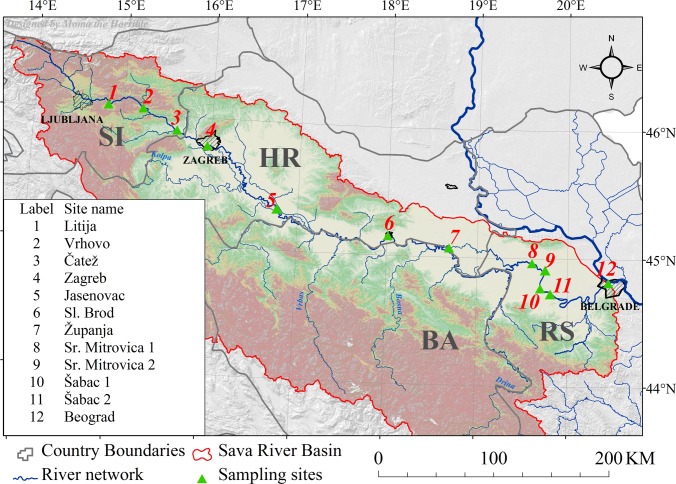
Sampling sites along the Sava River.

Sampling was performed in August and September 2015 at 12 sites along the river. Position of the sites is indicated in the Fig 1, while the list of analyzed parameters is summarized in Table 1.

**Table 1:** Overview of the parameters analyzed within the Sava survey 2015.

Parameter	Faecal indicators	SOS/*umuC*	Genotoxicity (standard and Fpg -comet assay and micronucleus)		Metals and metalloids		Condition Factor (mean ± SE)
Media	water	water	fish	N of specimens	fish	N of specimens	fish
Litija	+	+	*A*. *bipunctatus*	8	x	x	0.80±0.07
Vrhovo	+	+	*A*. *bipunctatus+A*. *alburnus*	6+2	*A*. *bipunctatus*	8	0.66±0.03
Čatež	+	+	*A*. *bipunctatus*	4	*A*. *bipunctatus*	10	1.00±0.17
Zagreb	+	+	*A*. *alburnus*	7	*A*. *alburnus*	6	0.66±0.02
WWZ*	+	+	x	x	x	x	x
Jasenovac	+	+	*A*. *alburnus*	9	*A*. *alburnus*	9	0.63±0.02
S. Brod	+	+	*A*. *alburnus*	8	*A*. *alburnus*	5	0.64±0.01
Županja	+	+	*A*. *alburnus*	7	*A*. *alburnus*	7	0.68±0.02
S. Mitrovica1	+	+	*A*. *alburnus*	7	*A*. *alburnus*	7	0.67±0.02
WWSM*	+	+	x	x	x	x	x
S. Mitrovica2	+	+	*A*. *alburnus*	8	x	x	0.68±0.02
Šabac 1	+	+	*A*. *alburnus*	5	*A*. *alburnus*	5	0.89±0.09
WWŠ*	+	+	x	x	x	x	x
Šabac 2	+	+	*A*. *alburnus*	8	*A*. *alburnus*	8	0.70±0.02
Belgrade	+	+	*A*. *alburnus*	8	*A*. *alburnus*	10	0.66±0.02

x-not assessed

*WW-wastewater outlet at the site listed above

### Indication of presence of wastewaters–faecal indicators

From each site a sample of 500 mL of water was taken in sterile glass bottles and transferred to a laboratory at 4°C. For the quantification of *E*. *coli*, Defined Substrate Technology (DST) was used, which detects *E*. *coli* by enzymatic hydrolysis of specific substrates [15]. Briefly, two dilutions of water samples (1:10 and 1:1000) were prepared and Colilert-18 powder was added, stirred and left at room temperature for a couple of minutes to dissolve. Suspensions were poured into a Colilert Quanti-Tray 2000 and sealed. Incubation was carried out at 37°C for 24 h and quantification was performed by the Most Probable Number (MPN) result, based on the color change and fluorescence in 97 wells.

### Mutagenic potency of water samples—SOS/*umuC*

From each site a sample of 50 mL of water was taken and stored at -20°C. The SOS/*umuC* assay was applied on stored water samples filtrated through 0.2 μm pore size filters using the protocol described by Žegura et al. [16]. The overnight culture of *S*. *typhimurium* TA1535/pSK1002 was diluted 10 times with fresh TGA (tryptone, glucose and ampicilin medium) and incubated at 37°C for 1.5 h with aeration until the bacteria reached the exponential growth phase. Treatment was performed in microtiter plates by adding 180 μL of water sample, 20 μL of 10 x TGA and 70 μL of bacterial culture or in the case of metabolic activation 180 μL water sample, 20 μL 10 x TGA with cofactors and 70 μL of S9 bacterial culture mixture prepared as described in the ISO standards [16]. 4-Nitroquinoline (4-NQO, final concentration 0.5 μg/mL) was used as a positive control in experiments without metabolic activation while benzo(a)pyren (final concentration 10 μg/mL) was used in experiments with metabolic activation. Sterile bidistilled water was used as the negative control. The microtiter plate was incubated at 37°C for 2 h with aeration. After the treatment, the incubation mixture was then diluted 10 times with fresh TGA medium in new microtiter plates and incubated for a further 2 h. The bacterial growth rate was determined by measuring absorbance at 600 nm (OD_600_) at microtitar plate reader. ß-Galactosidase activity was determined after using o-nitrophenyl-ß-D-galactopyranoside (ONPG) as a substrate for 20 min at 25°C. Absorption was measured at 405 nm using a reference solution without bacteria. The bacterial growth rate was calculated using the following formula: G = sample OD600/control OD600. A growth ratio less than 0.75, that represents 25% inhibition of biomass was considered to be an indication of cytotoxicity.

Induction ratio (IR) was calculated by the formula: sample OD405/(control OD405 x G). An induction ratio 1.5 was taken as the threshold at which the sample was considered as genotoxic [17]. All treatments were performed in triplicates in three individual experiments.

### Determination of soluble concentrations of metals and metalloids in water

For the determination of the soluble element content in water samples from the Sava River, samples were filtered through a 0.45 μm filter and concentrations were determined by inductively coupled plasma mass spectrometry (ICP-MS). For the determination of the total Hg concentration, all water samples were analyzed to prevent losses of dissolved gaseous Hg during the filtrations. The results are given in S1 Table. The data revealed that the concentrations of elements in water are low.

### Fish specimen collection

Whole body metal content and genotoxicological parameters were assessed in specimens of spirlin and bleak based on the on site species availability as indicated in Table 1.

All animal procedures were in compliance with Directive 2010/63/EU on the protection of animals used for experimental and other scientific purposes, and were approved by the Ethical Committee for the Use of Laboratory Animals of the Institute for Biological Research "Siniša Stanković", University of Belgrade. Fish sampling was approved by the Ministry of the Environment and Spatial Planning of the Republic of Slovenia, Ministry of Agriculture of Republic of Croatia and Ministry of Agriculture and Environmental Protection of Republic of Serbia. Specimens were collected by combining electro-fishing (Aquatech DC electro fisher IG 1300, 2.6 kW, 80–470 V) and fishing nets (mesh size 1 cm). The condition factor of the collected specimens was calculated according to the formula: CF = W/L^3^ × 100, where W is weight (g) and L is total length of fish (mm) [18]. Immediately after sampling, fish specimens were anesthetized with clove oil prior to dissection.

### Assessment of concentration of metals and metalloids in fish tissue

Each individual separately was grinded in a Laboratory homogenizer Sterilmixer (International P.B.I. S.p.A.) and whole body composite (wbc) samples were weighed using an electronic balance (± 0.1 g) and stored at -20°C prior to analysis.

Wbc samples were submitted to the Analytical Chemistry Laboratory within the Institute of Chemistry at the Faculty of Chemistry, University of Belgrade, Serbia, for chemical analysis. The element concentrations (Al, As, Cd, Co, Cr, Cu, Fe, Hg, Mn, Ni, Pb, Se, Sn and Zn) were determined by inductively coupled plasma optical emission spectrometry (ICP-OES), using a Thermo Fisher Scientific iCAP 6500 Duo ICP (Cambridge, United Kingdom) equipped with a RACID86 Charge Injector Device (CID) detector, concentric type nebulizer, quartz torch, and alumina injector. The following wavelengths of the ICP-OES analysis were used (nm): Al 394.401, As 189.042, Cd 228.802, Co 228.616, Cr 205.552, Cu 324.754, Fe 259.941, Hg 184.950, Mn 259.373, Ni 231. 604, Pb 220.353, Se 196.090, Sn 246.161 and Zn 206.191. The limits of detection (LOD) were: Al 0.076, As 0.013, Cd 0.028, Co 0.65, Cr 0.081, Cu 0.19, Fe 0.16, Hg 0.016, Mn 0.034, Ni 0.15, Pb 0.56, Se 0.12, Sn 0.54, Zn 0.057 μg/L. The limits of quantification (LOQ) were Al 0.381, As 0.045, Cd 0.091, Co 0.95, Cr 0.029, Cu 0.67, Fe 0.54, Hg 0.063, Mn 0.115, Ni 0.59, Pb 1.78, Se 0.45, Sn 1.64, Zn 0.180 μg/L.

Fish samples (~1.5 g) were dried in a lyophilizer (Christ Alpha 2–4 LD, Harz, Germany), and then digested in an Advanced Microwave Digestion System (ETHOS 1, Milestone, Italy) using a mixture of 65% nitric acid and 30% hydrogen peroxide (Suprapur®, Merck, Darmstadt, Germany, 10:2, v/v) at 200°C for 20 min. After cooling to room temperature and without filtration, the solution was diluted to a fixed volume (volumetric flask, 25 ml) with ultra-pure water with a conductivity of 0.055 μS/cm (Barnstead™ GenPure™ Pro, Thermo Scientific, Germany), before being analyzed by ICP-OES.

Blanks with no fish tissue were run with each batch of samples to monitor contamination by the reagents used. The standards for the instrument calibration were prepared on the basis of the multi-element (SS-Low Level Elements ICV Stock, 10 mg/L) and mono-element (Hg Calibration Stock, 10 mg/L Hg; Sn Calibration Stock, mg/L Sn) certified reference solutions ICP Standard (VHG Labs, Inc-Part of LGC Standards, Manchester, NH 03103 USA) and analyzed to support quality assurance and control. The muscle standard reference material (DORM-4; National Research Council of Canada) was digested in triplicate and analyzed to support quality assurance and control.

Mean values and standard deviations were calculated for each group and elements concentrations were expressed as mgkg^-1^ wet weight (ww). Kolmogorov-Smirnov test was used to determine if data were normally distributed. Differences in concentrations of elements in fishes from different sampling stations were analyzed by non-parametric Kruskal-Wallis test. Post hoc inter-group comparisons of element levels were performed by the Dunn’s Multiple Comparison Test. Statistical analysis of data was carried out using SPSS 16.0 statistical package programs for Windows (SPSS Inc., Chicago, IL, USA). The metal pollution index (MPI) was calculated to compare the total metal content in the different sampling stations using the following equation [19]: MPI = (cf1 x cf2 x cf3 x …cfn)^1/n^, where cf_n_ = concentration of the metal n in the sample.

### Blood sample collection and preservation

Blood samples were cryopreserved based on methodology described in Akcha et al. [20] with slight modifications. Blood was collected directly from the heart with 3 mL syringes (21 G needle rinsed with sodium heparin) and one drop of blood of each specimen was diluted 20x in 4°C cooled medium (RPMI 1640 supplemented with 25% fetal bovine serum). Due to storage limitations, blood samples from two specimens were pooled together to form a single sample. To all samples, cryoprotective agent was added (DMSO final concentration 20%) and samples were immediately frozen in liquid nitrogen until the analysis (up to 3 weeks).

In preliminary experiments, the effects of cryopreservation on cell viability and the level of DNA damage were assessed in 8 specimens of bleak collected at the site situated in the Sava River near Belgrade prior to Sava River survey.

### Comet assay

Samples were taken from liquid nitrogen and immediately thawed at 21°C in water bath. Cell viability was assessed by acridine orange/ethidium bromide differential staining described in details in Gačić et al. [21]. Afterwards, samples were diluted in 1xPBS to obtain approximately 50,000 cells/mL. The suspensions were centrifuged (2,000 rpm, 10 min, 4°C), the supernatants were discharged and pellets were suspended in 100 μL of residual supernatant. For each sample three slides were prepared: one for alkaline and two for Fpg–modified comet assay.

The comet procedure was performed under yellow light as described in Kolarević et al. [22] with slight modifications. Briefly, microscope slides were pre-coated with 1% normal melting point (NMP) agarose and air dried for 24 h. The second, supportive layer was formed of 80 μL of 1% NMP agarose. The final layer was formed of 30 μL of cells suspension (prepared as described earlier) gently mixed with 70 μL of 1% low melting point agarose (37°C). The slides were held in freshly made cold (4°C) lysis buffer (2.5 M NaCl, 100 mM ethylenediaminetetraacetic acid disodium salt dyhidrate (EDTA), 10 mM Tris, 10% dimethyl sulfoxide (DMSO), 1.5% Triton X-100, pH 10) for 2 h. To allow DNA unwinding, slides were placed in an electrophoresis chamber containing cold (4°C) alkaline electrophoresis buffer (300 mM NaOH, 1 mM EDTA, pH 13) for 20 min. Electrophoresis was performed with a voltage gradient 0.75 V/cm and amperage 300 mA for 20 min at (4°C). Afterwards, neutralisation was carried out in freshly made cold (4°C) neutralizing buffer (0.4 M Tris, pH 7.5) for 15 min. Slides were preserved by fixation in cold methanol at 4°C for 15 min. Staining was performed with 20 μL per slide of acridine orange (2 μg/mL). The slides were examined with a fluorescence microscope (Leica, DMLS, Austria, under magnification 400 X, excitation filter 510–560 nm, barrier filter 590 nm). Microscopic images of comets were scored using Comet IV Computer Software (Perceptive Instruments, UK). Tail intensity (TI %—percentage of DNA in the tail of the comet) was chosen as a measure of DNA damage. For each sample 100 nucleoids were scored. As the possible indication of apoptosis, excessively damaged nuclei or so called hedgehogs (HH) were counted for each slide using an hedgehog tool available in the Comet IV Software.

### Fpg–modified comet assay

For each sample, two slides were prepared for Fpg—modified assay as described in section 2.8, one for buffer and one for the enzyme. After one hour of lysis slides were washed 3 times in cold (4°C) washing buffer (100 mM KCl 100 mM, 10 mM Na_2_EDTA and 10 mM HEPES, adjusted pH 7.2). At slides prepared for buffer, 45 μL of buffer (100 mM KCl, 10 mM EDTA, 10 mM HEPES, 0.1 mg/mL BSA, adjusted pH 7.2) was added while on slides prepared for enzyme 45 μL of 300 x diluted Fpg enzyme (Trevigen, Maryland) was added and covered with coverslips. Slides were incubated for 30 min at 37°C in humidity chamber. Afterwards slides were held for 5 min at 4°C, coverslips were removed and slides were subjected to denaturation step as for the standard comet assay protocol described in the section 2.7. The net contribution of the 8-hydroxy-2′-deoxyguanosine (8-oxoG) in final DNA damage evaluated by Fpg—modified comet assay was calculated by subtraction of the mean TI% values obtained from slides exposed to buffer only from the mean TI% values obtained from the slides exposed to Fpg enzyme [23].

### Micronucleus assay

Slides for micronucleus assay were prepared as described in Štraser et al. [24], air dried for 24 h, stained with acridine orange 25 μg/mL and examined at 1000x magnification. For each sample at least 3,000 cells was examined. Nuclear aberrations were scored by criteria of Fenech [25]. Diameter for micronuclei was between 1/3 and 1/16 of the main nuclei.

### Statistical analyses of data in gentoxicological bioassays

Statistical analysis of the results obtained in the experiments was carried out using Statistica 6.0 Software (StatSoft, Inc.) and SPSS 20.0 (Inc., Chicago, IL, USA). Kolmogorov-Smirnov test was used to determine if data were normally distributed. Data on MN frequency were analyzed by one-way ANOVA followed by Tukey’s post-hoc test. Comet assay data were analyzed by Kruskal-Wallis one-way ANOVA followed by Dunn’s Multiple Comparison Test since they were not normally distributed. The level of significance for all comparisons was set at p < 0.05. Correlation analyses were carried out using Pearson’s correlation test with significance level p < 0.05.

### Ranking of the sites by integrated biomarker response (IBR)

The IBR ranking of the sites was performed based on parameters–*E*. *coli* numbers (EC), metal pollution index (MPI), condition factor (CF), mutagenicity (SOS with metabolic activation), comet assay (CA), oxidative stress approximated with net contribution of 8-oxoG sites (OS) and micronucleus assay (MN). IBR was assessed as described by Beliaeff and Burgeot [26]. Briefly, the value of each parameter (X_i_) was standardized by the formula Y_i_ = (X_i_ -mean)/SD, where Y_i_ is the standardized parameter response, mean and SD are calculated based on values for the selected parameter for all sites. Z_i_ was then calculated as Z_i_ = Y_i_ if the studied parameter respond to contamination by induction or Z_i_ = -Y_i_ if the parameter respond to contamination by inhibition. The minimum value Z_i_ for each parameter was marked (min) and the scores for the studied parameters were computed as S_i_ = Z_i_ + |min|. Scores for each parameter (S_i_) for particular site were used as radius coordinates of the studied parameters of the star plots. Individual areas Ai of the star plot were calculated according to the formula: Ai = S_i_ x S_i+1_ x sin (51.43°) / 2, where S_i_ and S_i+1_ represent the individual parameter scores and their successive star plot radius coordinates. The IBR value is calculated as following: IBR = sum of all A_i_, where A_i_ is the area represented by two consecutive indicators on the star plot, and n is the number of indicators used in the IBR calculation. Scores for each parameter (S_i_) were used for ranking of the parameters while the IBR values were used for final ranking of the sites. The site with the lowest rank was considered as the site with the lowest level of stressors.

## Results

### Indication of presence of wastewaters

Numbers of *E*. *coli* were assessed in river water samples and samples collected at wastewaters discharge points (S1 Fig). Majority of river samples were slightly and moderately polluted. The highest numbers of *E*. *coli* were recorded in a river sample collected at the site Županja (32,300 MPN/100 mL). Wastewaters discharge points were found at the sites Zagreb (WWZ), S. Mitrovica (WWSM) and Šabac (WWS). Samples collected from the WW discharge points at the sites S. Mitrovica and Šabac indicated excessive faecal pollution affecting water quality at downstream situated sites (S. Mitrovica 2 and Šabac 2 respectively). Therefore, the sites Županja, S. Mitrovica 2 and Šabac 2 are recognized as hotspots of faecal pollution.

### SOS/*umuC*

**Fig 2:**
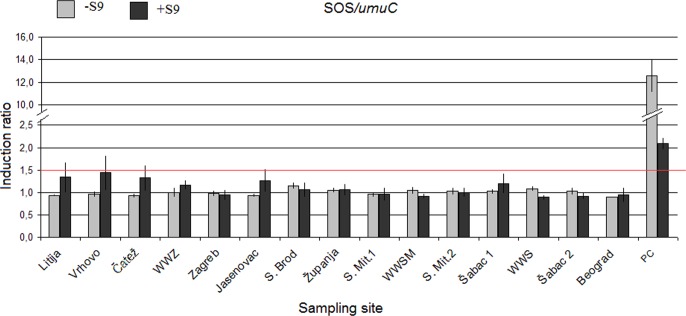
SOS induction rate (mean ± SE) in SOS/*umuC* assay. Red line represents threshold induction value (1.5); PC—positive controls: 4NQO (0.5 μg/mL) in experiments without metabolic activation and benzo(a)pyrene (10 μg/mL) in experiments with metabolic activation.

The results are summarized at the Fig 2. In investigated water samples cytotoxic effect were not recorded and threshold value 1.5 of induction was not breached in any case. The highest induction was detected at the sites Litija and Vrhovo. Positive controls for experiment (4NQO and benzo(a)pyrene) affirmed validity of experimental system.

### Assessment of concentration of metals and metalloids in fish tissue

During the chemical analysis of metals and metalloids, a replicate analysis of the reference material showed good accuracy, with recovery ranging from 89.80% to 103.75%. The average concentrations in wbc samples of spirlin and bleak and MPI values are presented in Table 2.

**Table 2:** The average element concentrations in whole body composite (wbc) samples of *A*.*alburnus* and *A*. *bipunctatus* (marked with *) in mgkg^-1^ ww, and MPI values for each sampling station; different letters denote significant differences among studied sites (p < 0.05).

Sampling station/Elements	Vrhovo *	Čatež *	Zagreb	Jasenovac	Slavonski Brod	Županja	Sremska Mitrovica	Šabac 1	Šabac 2	Belgrade
Al	5.49 ± 5.72^ab^	3.31 ± 0.18^a^	2.92 ± 2.42^ab^	4.33 ± 3.26^ab^	2.08 ± 0.88^ab^	21.12 ± 28.08^b^	13.49 ± 19.83^ab^	5.83 ± 3.76^ab^	10.15 ± 10.94^b^	1.79 ± 0.93^ab^
As	0.008 ± 0.011^a^	0.15 ± 0.06^b^	0.12 ± 0.025^b^	0.053 ± 0.037^ab^	0.13 ± 0.09^ab^	0.067 ± 0.059^ab^	0.051 ± 0.048^ab^	0.066 ± 0.069^ab^	0.065 ± 0.036^ab^	0.1 ± 0.086^ab^
Cd	0.008 ± 0.004^ab^	0.008 ± 0.002^ab^	0.1 ± 0.002^b^	0.007 ± 0.001^ab^	0.016 ± 0.008^b^	0.002 ± 0.002^a^	0.0018 ± 0.0016^a^	0.001 ± 0.001^a^	0.008 ± 0.007^ab^	0.013 ± 0.008^b^
Co	0.0009 ± 0.002^ab^	0.0007 ± 0.0006^ab^	0.001 ± 0.0007^ab^	0.0001 ± 0.0004^a^	0.0008 ± 0.0004^ab^	0.02 ± 0.03^b^	0.009 ± 0.016^ab^	0.002 ± 0.004^ab^	0.003 ± 0.007^ab^	0.0006 ± 0.0004^ab^
Cr	0.35 ± 0.13^a^	0.17 ± 0.022^b^	0.18 ± 0.045^ab^	0.224 ± 0.06^ab^	0.154 ± 0.024^b^	0.25 ± 0.093^ab^	0.198 ± 0.109^ab^	0.17 ± 0.024^ab^	0.18 ± 0.024^b^	0.18 ± 0.02^b^
Cu	0.9 ± 0.33^ac^	0.48 ±0.05^bd^	0.67 ± 0.12^cd^	0.61 ± 0.15^abcd^	0.68 ± 0.34^abcd^	0.8 ± 0.39^abcd^	0.76 ± 0.38^abcd^	0.49 ± 0.072^abcd^	0.78 ± 0.39^abcd^	0.44 ± 0.14^b^
Fe	21.5 ± 11.52^a^	9.74 ± 3.2^ab^	14.5 ± 8.27^ab^	13.79 ± 5.05^ab^	11.44 ± 6.67^ab^	27.12 ± 25.45^ab^	19.08 ± 19.36^ab^	11.01 ± 3.43^ab^	14.88 ± 9.26^ab^	8.76 ± 3.44^b^
Hg	0.08 ± 0.35^ad^	0.02 ±0.004^cd^	0.013 ± 0.003^bc^	0.102 ± 0.059^a^	0.011 ± 0.003^bc^	0.028 ± 0.014^abcd^	0.027 ± 0.019^abcd^	0.041 ± 0.01^abcd^	0.037 ± 0.027^abcd^	0.011 ± 0.004^bc^
Mn	4.63 ± 2.49^ae^	1.37 ± 0.48^bc^	1.55 ± 0.71^abcde^	2.85 ± 0.89^abcde^	1.2 ± 0.47^bd^	5.34 ± 3.4^ae^	4.78 ± 3.73^ade^	3.37 ± 1.20^abcde^	4.79 ± 2.9^e^	1.73 ± 0.72^acd^
Ni	0.03 ± 0.018^a^	0.02 ± 0.004^abc^	0.032 ± 0.033^abc^	0.027 ± 0.013^abc^	0.034 ± 0.01^abc^	0.155 ± 0.137^b^	0.126 ± 0.096^b^	0.047 ± 0.033^abc^	0.06 ± 0.03^dc^	0.025 ± 0.015^ac^
Pb	0.037 ± 0.042^abcd^	0.11 ±0.02^ad^	0.17 ± 0.03^abcd^	0.024 ± 0.027^bc^	0.1 ± 0.01^abcd^	0.057 ± 0.076^abce^	0.056 ± 0.06^abcd^	0.014 ± 0.014^cd^	0.077 ± 0.12^abcd^	0.11 ± 0.26^a^
Se	0.93 ± 0.17^a^	0.24 ±0.06^bc^	0.34 ± 0.066^ac^	0.27 ± 0.052^acd^	0.11 ± 0.025^bdf^	0.3 ± 0.06^acf^	0.27 ± 0.039^abcdef^	0.28 ± 0.021^abcdef^	0.28 ± 0.032^acf^	0.085 ± 0.04^b^
Sn	0.028 ± 0.028^a^	0.016 ±0.0007^a^	0.016 ± 0.0008^a^	0.023 ± 0.032^a^	0.015 ± 0.002^a^	0.04 ± 0.058^a^	0.02 ± 0.013^a^	0.009 ± 0.003^a^	0.025 ± 0.026^a^	0.016 ± 0.002^a^
Zn	47.67 ± 9.73^a^	24.74 ± 7.2^b^	24.47 ± 6.6^b^	28.37 ± 6.26^ab^	23.48 ± 4.82^b^	36 ± 10.14^ab^	34.47 ± 5.98^ab^	35.42 ± 13.87^ab^	38 ± 11.48^ab^	23.09 ± 6.65^b^
MPI	0.23	0.17	0.23	0.16	0.16	0.33	0.26	0.16	0.26	0.14

A pattern was observed at all sampling sites with the highest Zn concentrations (23.09 ± 6.65 to 47.67 ± 9.73) and at almost all of the sampling sites with the lowest Co concentrations (0.0001 ± 0.0004 to 0.009 ± 0.016) (Table 2). The highest concentrations and numbers of metals (Al, Co, Fe, Mn, Ni and Sn) were recorded at Županja sampling site. On the other hand, the lowest concentrations of metals and metalloids (Al, Cu, Fe, Hg, Se, Zn) were in the highest number determined at the Beograd sampling station.

These results affected MPI values, and therefore the highest MPI was recorded for the Županja sampling station (0.33) and the lowest for the Belgrade sampling station (0.14). However, when comparing the values for concentrations of metals and metalloids at these two sites statistically significant difference was observed only for Cd and Ni.

When comparing the data of faecal pollution and data on concentration of metals and metalloids in fish tissue, significant correlation (r = 0.78, p = 0.008) was observed between the numbers of *E*. *coli* and MPI.

### Effects of cryopreservation and data validation

**Fig 3:**
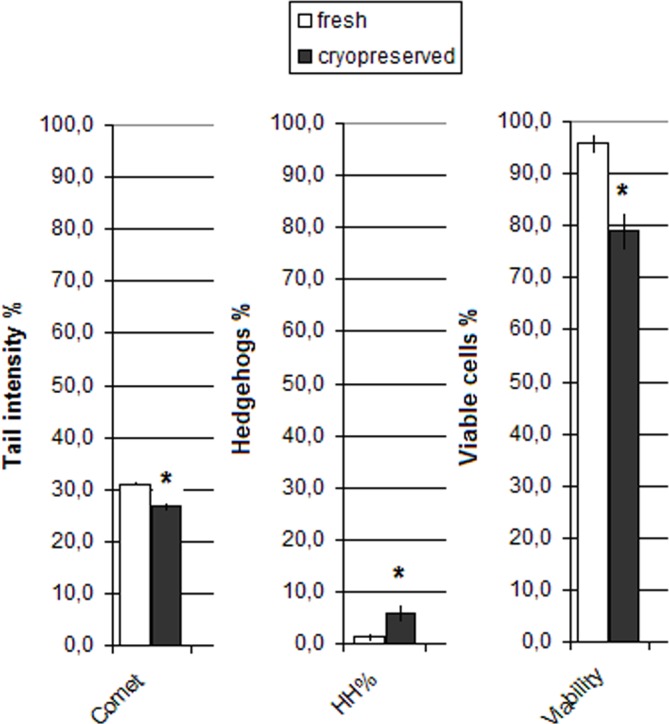
Cryopreservation effects on the cell viability and DNA damage level. *statistical significance in comparison with fresh sample (p < 0.05).

As indicated in Fig 3, the effects of cryopreservation on cell viability and DNA damage induction were assessed. Cell viability was reduced for 18% while cryopreservation did not additionally increased DNA damage level.

Although the values were within the same range, the level of DNA damage after cryopreservation was significantly lower comparing to the fresh sample. However, significant increase of HH frequency was noticed.

#### Assessment of cell viability in cryopreserved samples

Average cell viability in cryopreserved samples was about 80% (Table 3). The lowest viability was observed in a sample collected at the site Vrhovo (65 ± 6%) indicating possible cytotoxic effect. Significant negative correlation was observed between the cell viability and HH frequency (r = -0.61, p = 0.0361).

**Table 3:** Cell viability, frequency of hedgehogs and frequency of micronuclei in fish blood samples (mean ± SE).

Site	Viability %	HH%	MN‰
Litija	77 ± 2	5.5 ± 3.1	0.4 ± 0.1
Vrhovo	65 ± 6	10.8 ± 0.5	2.9 ± 1.4
Čatež	79 ± 3	2 ± 1.4	0.8 ± 0.5
Zagreb	84 ± 2	3.8 ± 1.1	3.4 ± 1.6
Jasenovac	71 ± 2	5.5 ± 2.7	0.3 ± 0.1
S. Brod	82 ± 3	0	0.8 ± 0.2
Županja	79 ± 3	3.3 ± 3.3	1.9 ± 0.3
S. Mitrovica 1	77 ± 7	12.8 ± 4.3	0.3 ± 0
S.Mitrovica 2	74 ± 3	4.3 ± 1.3	1.7 ± 0.7
Šabac 1	78 ± 4	10.5 ± 5.5	0.5 ± 0.2
Šabac 2	85 ± 1	0.3 ± 0.3	0.3 ± 0.1
Belgrade	87 ± 1	3.3 ± 1.2	0.5 ± 0.2

### Assessment of the DNA damage in blood cells

**Fig 4:**
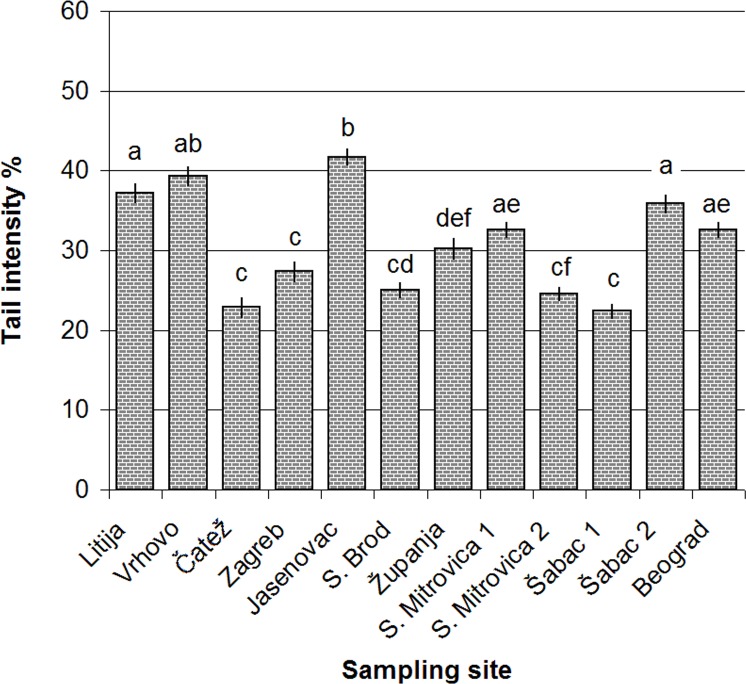
The values of tail intensity % obtained in standard alkaline comet assay in fish blood cells; values are represented as mean ± SE; different letters denote significant differences among studied sites (p < 0.05).

Alkaline comet assay was performed for the assessment of DNA damage while Fpg -modified comet assay was performed for the assessment of oxidative stress. Data obtained by alkaline comet assay indicated variation of DNA damage within the studied sites (Fig 4). The sites with the highest levels of DNA damage were Litija, Vrhovo, Jasenovac and Šabac 2. Impact of wastewater discharges was evident at the site Šabac 2 which had significantly higher TI% values in comparison with upstream situated Šabac 1.

Relation between the data obtained in the standard comet assay, HH% and cell viability was investigated. There was neither significant correlation between the level of DNA damage and HH% (r = 0.20; p = 0.53) nor DNA damage and cell viability (r = -0.36; p = 0.25).

### Fpg–modified comet assay

**Fig 5:**
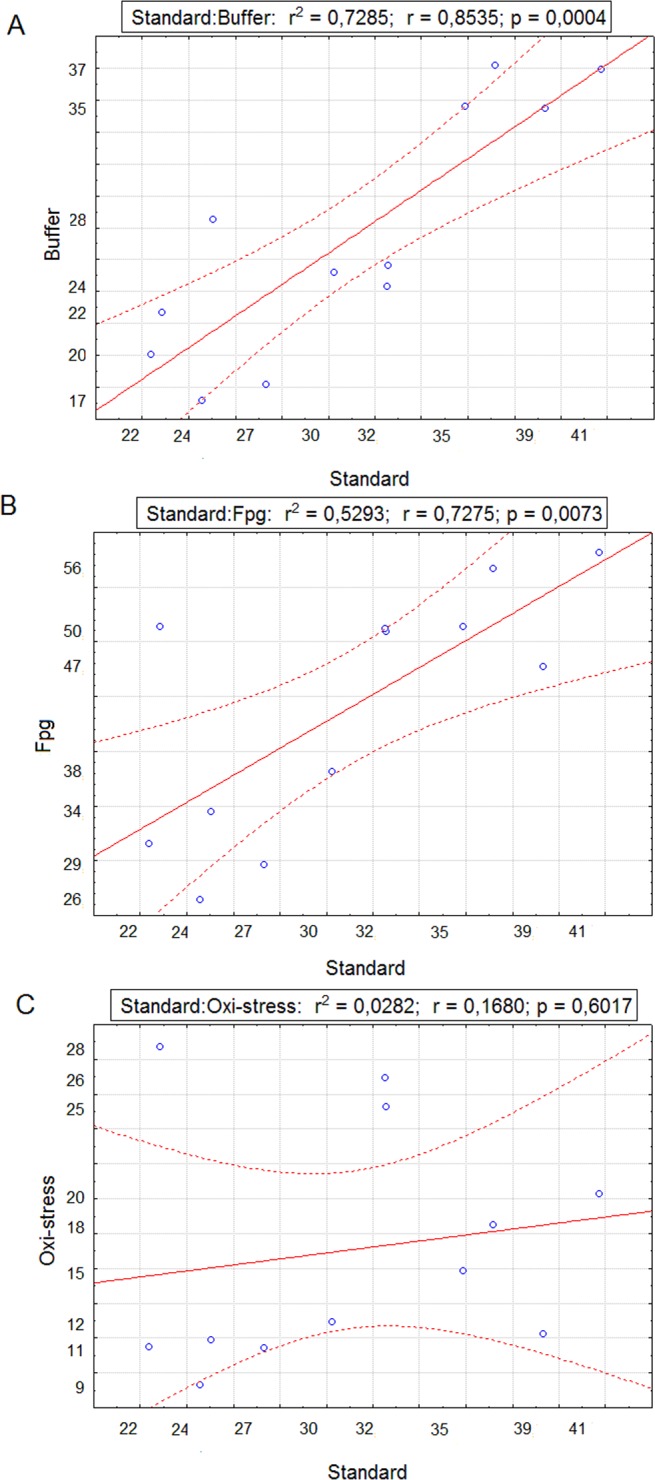
Correlation between the levels of DNA damage obtained in different assays. Correlation of the values obtained in standard comet assay and (A) buffer exposed slides, (B) Fpg exposed slides and (C) net 8-oxoG sites; full line—regression line; dashed line—95 confidence level.

Data obtained in Fpg—modified assay are summarized in the Table 4. TI% values from slides exposed to buffer only were within the same range as values in the standard alkaline comet assay, moreover significant positive correlation between the values was observed (Fig 5A).

**Table 4:** Fpg—modified comet assay, tail intensity values for buffer and Fpg—exposed slides and net contribution of 8-oxoG sites (mean ± SE); different letters denote significant differences among studied sites (p < 0.05).

Site	Fpg enzyme	Buffer only	Net 8-oxoG
Litija	56.7 ± 1.2^ad^	38.2 ± 1.2^a^	18.5
Vrhovo	47.7 ± 1.2^b^	35.5 ± 1.1^a^	12.2
Čatež	51.4 ± 1.6^ab^	22.7 ± 1.3^b^	28.7
Zagreb	29.6 ± 1.2^cf^	18.2 ± 1.0^c^	11.4
Jasenovac	58.1 ± 1.1^d^	37.9 ± 1.1^a^	20.2
S. Brod	34.4 ± 1.0^ce^	28.5 ± 1.0^d^	6.0
Županja	38.1 ± 1.2^e^	25.2 ± 1.3^bd^	12.9
S. Mitrovica 1	50.9 ± 1.1^ab^	25.6 ± 1.1^bd^	25.3
S. Mitrovica 2	26.4 ± 1.0^f^	17.2 ± 0.9^c^	9.3
Šabac 1	31.5 ± 1.0^cef^	20.0 ± 1.0^bc^	11.5
Šabac 2	51.4 ± 1.2^ab^	35.6 ± 1.1^a^	15.8
Belgrade	51.2 ± 1.3^ab^	24.3 ± 1.1^b^	26.9

In all cases digestion with Fpg enzyme led to significant increase of DNA damage in comparison with corresponding standard alkaline and buffer treated control. Significant correlation was observed between the mean TI% values obtained from slides for standard alkaline comet assay and slides exposed to Fpg enzyme (Fig 5B). The highest net contribution of 8-oxoG sites was detected in specimens from Čatež, S. Mitrovica 1 and Belgrade (Table 4). When plotting the values of TI% obtained in the standard alkaline comet assay and the values on net contribution of 8-oxoG sites in DNA damage, significant correlation was not observed (Fig 5C).

### Micronucleus

The highest frequency of MN was detected in specimens collected at the sites Vrhovo and Zagreb (Table 3). However, in comparison with the reference site there was no significant difference in MN frequency at any of investigated sites.

### Correlation of genotoxicological parameters and concentrations of metals in fish

Significant positive correlation was observed only between the values of TI% in alkaline comet assay and concentration of Hg in tissue (r = 0.75, p = 0.013). Significant negative correlation was observed between the frequency of 8-oxoG sites and concentration of Cu (r = -0.66, p = 0.038) (Table 5).

**Table 5:** Correlation between the monitored genotoxicological parameters and concentration of metals and metalloids in fish tissue; marked correlations are significant (p < 0.05).

Assay	parameter	Al	As	Cd	Co	Cr	Cu	Fe	Hg	Mn	Ni	Pb	Se	Sn	Zn
Comet	r	0.00	-0.60	-0.12	-0.13	0.62	0.39	0.27	**0.75**	0.32	-0.17	-0.31	0.44	0.51	0.38
	p	0.993	0.066	0.751	0.730	0.057	0.268	0.453	0.013	0.363	0.642	0.389	0.202	0.129	0.278
Fpg—comet	r	-0.26	-0.09	-0.32	-0.33	-0.23	-0.17	-0.22	0.50	-0.08	-0.45	-0.15	0.09	0.22	0.00
	p	0.472	0.814	0.360	0.347	0.526	0.643	0.550	0.145	0.836	0.192	0.677	0.815	0.546	0.987
8-oxoG sites	r	-0.40	0.44	-0.17	-0.34	-0.22	**-0.66**	-0.54	-0.05	-0.48	-0.47	0.23	-0.33	-0.16	-0.48
	p	0.252	0.202	0.634	0.340	0.543	0.038	0.105	0.893	0.159	0.171	0.524	0.354	0.659	0.161
MN	r	0.13	-0.15	0.62	0.19	0.51	0.54	0.54	-0.06	0.13	0.16	0.34	0.62	0.27	0.27
	p	0.726	0.673	0.055	0.600	0.135	0.108	0.109	0.870	0.720	0.665	0.339	0.058	0.455	0.454

### Ranking of the sites by IBR

**Fig 6:**
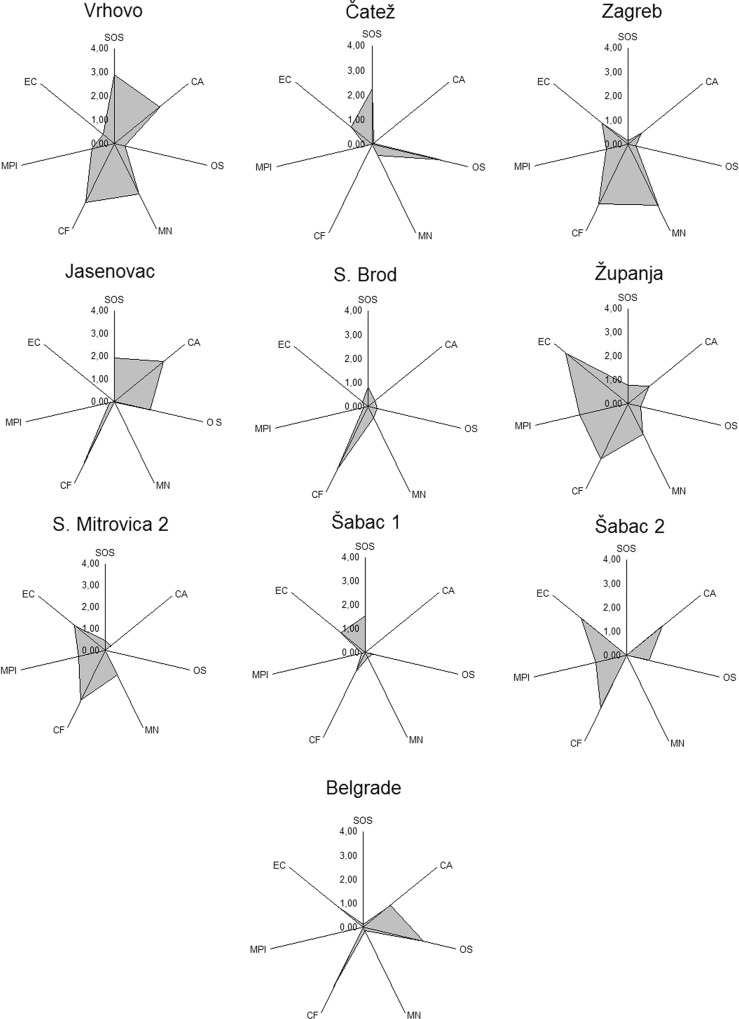
Graphical presentation of IBR.

The final IBR values as well as ranking for each studied marker are graphically presented at Fig 6. The site Šabac 1 had the lowest IBR rank (1.03) while the Županja and Vrhovo had the highest ranks (8.15 and 7.97 respectively) (Table 6).

**Table 6:** Integrated Biomarker Response (IBR) ranking of the studied sites.

	Vrhovo	Čatež	Zagreb	Jasenovac	S. Brod	Županja	S. Mitrovica 2	Šabac 1	Šabac 2	Belgrade
SOS/umuC (+S9)	2.87	2.23	0.14	1.92	0.78	0.78	0.46	1.54	0.00	0.12
CA	2.44	0.07	0.71	2.78	0.38	1.13	0.32	0.00	1.94	1.46
OS	0.43	2.82	0.32	1.59	0.38	0.53	0.00	0.32	0.95	2.57
MN	2.36	0.52	2.80	0.02	0.53	1.43	1.25	0.22	0.00	0.18
CF	2.75	0.00	2.75	2.98	2.93	2.59	2.56	0.87	2.45	2.78
MPI	0.96	0.30	0.92	0.23	0.18	2.08	1.27	0.15	1.33	0.00
EC	0.59	1.11	1.38	0.00	0.29	3.35	1.79	1.33	2.45	1.41
**IBR**	**7.97**	**1.80**	**5.03**	**4.11**	**1.17**	**8.15**	**3.78**	**1.03**	**3.25**	**1.98**

## Discussion

### General

In this study we have performed comprehensive genotoxicological survey by applying the battery of assays in prokaryotes and aquatic eukaryotes. When constructing the battery of bioassays we were focused on types of assays which have already been employed earlier in ecogenotoxicological studies of the Sava River [8–14, 27]. Also, we have used experience gained during the Joint Danube Survey 3 –JDS3 [22, 28, 29, 30] for updating the assay list as usage of the same methodology would enable comparison of water quality of the Sava and Danube in further research. As the assays employed in the study showed different sensitivity in detection of the stressors at investigated sites we have finally employed IBR approach to identify the most critical spots on the river but also to recommend which site has potential to be a reference site for Sava River. As the reference site is defined as the site which is minimally exposed to the stressors of interest [31], the Šabac 1 was chosen as the best available option.

### Indication of presence of wastewaters

The whole Sava River Basin is receiving high amounts of untreated or improperly treated wastewaters originating from the various size settlements that lie on the banks of the Sava River and its tributaries [1]. Therefore, our primary goal was identification of hotspots of contamination with wastewaters as possible sources of genotoxic pollution. As an indication of wastewater effluents, we have decided to use the numbers of *E*. *coli* in water. The results of the JDS3 confirmed that this parameter and concentration of caffeine in the Danube River are the most reliable indicators of contamination related to wastewaters [28, 32]. Indeed, wastewater outlets were recorded at three sampling sites (Zagreb, S. Mitrovica and Šabac), but based on concentrations of *E*. *coli*, throughout the full river course, only two hotspots of faecal contamination (critical faecal pollution) were identified (Županja and Šabac 2).

### Mutagenicity of water samples

The SOS/*umuC* was included in our research as the study of Žegura et al. [16] and Kittinger et al. [6] indicated the assays high potency in detection of genotoxic potential in wastewaters and surface waters. We have assumed that the assay would provide preliminary screening of genotoxic potential, but the induction ratio has not exceeded value 1.5 (threshold value) in any of the investigated samples. Similarly, in the study of Kittinger et al. [30] performed on the water samples of the Danube River, only four sites out of 68 investigated within the JDS3 showed mutagenic potential, indicating either to low sensitivity of the assay or low genotoxic potential of water.

### Indication of possible industrial pollution

In the moment of sampling, concentrations of metals and metalloids in water at the sampling sites were generally low. Therefore we focused on the concentrations of the elements accumulated in the tissues of selected species as an indication of prolonged exposure. Recent studies regarding metals and metalloids contamination in the Sava River were obtained in the European chub (*Squalius cephalus*) [33–35]. Based on the dataset obtained in our earlier survey of the Sava River [36], we have selected two minnow species, which are ecological equivalents in different communities. Spirlin is characteristic for the upper section and bleak is present in middle and lower sections of the river. Both spirling and bleak are active and fast swimmers, of the short life-span, with consequently high metabolic rates which can lead to high accumulation of metals and metalloids. Also, they are both epipelagic fish, not exposed to the contaminants in the benthic zone. Based on these features, bleak was successfully used for estimation of metals and metalloids in studies of Uysal et al. [37], Al Sayegh Petkovšek et al. [38], Mercai et al. [39] and many others.

We estimated the highest industrial pollution with the highest observed MPI for the Županja sampling site and of lowest for the Belgrade sampling site. This result is in accordance with Dragun et al. [40] who previously reported that Sava River water reflects a certain anthropogenic impact in the Croatian section of the river while, on the other hand, Vuković et al. [41] stated that the industrial activity in Serbia slowed down during the past two decades, which is the reason for the weakly noticeable anthropogenic input of heavy metals in the Sava River system (water and sediment) from the nearby environment.

Although we have assessed the presence of wastewaters by two aspects (indicators of faecal pollution and industrial pollution) it is important to emphasize that significant correlation was observed between the numbers of *E*. *coli* and MPI clearly showing that at the majority of the sites pollution from a single source prevails.

When making the parallel of the data on metal accumulation in tissue and the data from genotoxicological assays, we would emphasize that the positive correlation was observed between the concentration of mercury and TI% values. Mercury is listed as a priority substance by Directive 2008/105/EC [42] whose genotoxic potential in aquatic environment is well known [43]. Negative correlation was detected between the oxidative stress and concentration of copper which is understandable as copper can have a protective effect as a constitutive of superoxide dismutase [44]. Correlations which were not significant were not taken into consideration because of the possible causative effect.

### Assessment of genotoxic potential along the river

#### An overview of the selected bioassays

The dataset obtained in our study represents only the so called snapshot of the current status of the river; therefore, we have chosen bioassays which would enable detection of the effects of recent genotoxic pollution and the effects of the prolonged exposure to pollution as well. As the SOS/*umuC* provides only information on genotoxic potential of water sampled in particular moment, we have decided to evaluate the effect of prolonged exposure in aquatic animals inhabiting the studied sites. In our previous research we have shown that various fish species can be used as reliable bioindicators for the detection of genotoxic pollution [45–47]. In our current study spirlin and bleak were the most convenient option due to reasons indicated before and moreover, during the JDS3 we managed to cover over 2,000 rkm of the Danube with bioassays in blood of bleak [29].

Comet assay was employed to detect DNA damage occurred recently; this method is the most commonly used for assessment of pollution related genotoxicity in aquatic organisms [48]. We have introduced Fpg—modified comet assay which additionally detects the DNA damage caused by oxidative stress in specimens as the oxidative stress is identified as the major contributor to DNA damage in the majority of studies dealing with aquatic environments. Generally, the mode of action of priority substances in environment is based on generation of reactive oxygen species [49]. As we have not observed correlation between the standard comet assay and frequency of 8-oxoG sites we can speculate that oxidative stress is not the only or major contributor of the detected gentoxic potential. Finally, micronucleus assay was used for the detection of permanent damage indicating presence of potential clastogenic and/or aneugenic agents. Consequently, the standard and Fpg—modified comet assay showed higher potential in differentiation of the sites based on genotoxic potential in comparison with micronucleus assay and SOS/*umuC* test.

#### Cryopreservation of blood samples and data validation

As the fish blood samples could not be analyzed directly on site, we have decided to perform the comet assay on samples cryopreserved by immediate immersion in liquid nitrogen. This kind of sample processing is indicated as reliable for assessment of DNA damage by numerous studies [50–52]. Prior to the survey we have performed preliminary analyses to investigate if the cryopreservation can influence the comet assay results in the blood samples of bleak. The results indicated that cryopreservation reduces cell viability while the level of DNA damage was within the same range as in the fresh sample which is in compliance with the results of Akcha et al. [20]. However, considering that cryopreservation affects cell viability, we wanted to investigate possible interference of apoptosis on the comet assay results in samples collected during survey. The significant correlation was observed between the level of cell viability and hedgehogs frequency, which was expected as by some authors cyototoxicity is manifested in comet assay by appearance of highly damaged nuclei–so called ghosts or hedgehogs [23, 53, 54]. However, it is important to emphasize that TI% values obtained in comet assay did not show a correlation with the cell viability or the frequency of hedgehogs and therefore data on these parameters were studied independently.

#### Genotoxic potential along the river and overview of the literature data

**The upper Sava (Slovenia):** In the Slovenian stretch of the river, the highest genotoxic potential was detected at the site Vrhovo. At this site, we have observed a significant increase of TI% values in comparison to the reference site accompanied with the highest MN frequency. Increased induction ratio in SOS/*umuC* also points to possible mutagenicity. This site was not identified as a hotspot of faecal pollution, but we have noticed metal contamination by the highest concentrations of Cr, Cu and Zn in fish tissue, when compared to other sites. Moreover, the IBR rank of the site was among the highest. The study of Källqvist et al. [55] indicated that pore-water samples from the site Vrhovo had several fold higher toxic effect (algae growth inhibition test) in comparison with other sites on the Sava River. Moreover, the study of Milačič et al. [56] indicated that sediment at this site contains high concentrations of metals (Ni, Zn, Cu, Cd) which can have considerable genotoxic potential [57].

Going further downstream, the situation is completely different at the site Čatež. In comparison with the site with the lowest IBR rank (Šabac 1), there was no difference in the level of DNA damage measured by micronucleus or comet assay. This site caught our attention as a possible reference site on the basis of available genotoxicological literature data. As mentioned before, the section of the river stretch from the Slovenian-Croatian border to the confluence of the Una River is the most studied part. In the study of Pavlica et al. [11] the area nearby the site Čatež (few km downstream) was used as a reference site for the assessment of genotoxicity along the Sava River by bioassays performed in European chub (*Squalius cephalus*). The same group of authors also confirmed low level of genotoxic pollution in the mentioned area in their study performed on zebra mussel *Dreissena polymorpha* [8]. However, at this site we have detected the highest level of oxidative stress which can be linked to the highest concentration of As measured in tissue [56].

**The middle Sava (Croatia):** Zagreb (750,000 inhabitants) is the largest urban settlement situated in the middle stretch of the Sava River. In previous studies performed in the Croatian stretch of the river, major focus was placed on this site as a greatest source of pollution In the studies of Klobučar et al. [8, 9] and Pavlica et al. [11], presence of genotoxic pollution was detected by comet and micronucleus assays in various aquatic organisms (fish, crayfish, mussels) sampled in the industrial zone located downstream from the city. Our results indicated that environmental quality has improved when compared to the data obtained in the period prior to implementation of wastewater treatment facility. None of the applied bioassays has indicated increase of genotoxic potential in comparison to upstream situated Čatež. By the numbers of *E*. *coli*, this site was not recognized as a hotspot of faecal pollution, but still the highest concentrations of Cd and Pb in fish tissue were recorded there.

Surprisingly, among the studied sites, the highest values of TI% were detected at the site Jasenovac. The site is characterized by low level of faecal pollution but with the highest concentration of mercury in the fish tissue. In the middle section, the site with the highest rank by IBR value was Županja. The site was identified as hotspot of faecal pollution with the highest value of MPI (the highest concentration of Al, Fe, Ni, Mn, Se). Comparing to the site with the Šabac 1, significant increase of DNA damage measured by the standard comet assay was observed. Observed genotoxic potential is in compliance with data of our previous study performed on mussels (*Unio* sp.) on the site situated close to Županja (2 km upstream) [13].

**The lower Sava (Serbia):** In Serbian stretch we have investigated the impact of the largest settlements situated on the river banks: Sremska Mitrovica (40,000 inhabitants), Šabac (50,000 inhabitants) and Obrenovac (50,000 inhabitants). Data of our previous study indicated presence of genotoxic potential at these sites [13]. In this section, town Šabac had the highest impact on water quality. While the site situated upstream (Šabac 1) had the lowest IBR value among all studied sites, situation was quite different at the site downstream the wastewater outlets (Šabac 2) which was identified as a hotspot of faecal pollution with evident indications of genotoxic potential by both standard and Fpg—modified comet assay. The site Belgrade is situated upstream of the urban area of Belgrade city. This stretch is mainly impacted by upstream situated city Obrenovac and associated settlements. Previously we have demonstrated that the area is under pressure of genotoxic pollution which is reflected in animals belonging to different trophic levels. [12, 14]. At this site, we have detected a significant increase of DNA damage measured by TI%, and increased levels of oxidative stress in comparison with the reference site, which is in compliance with the data on genotoxicity previously obtained in bream species [14].

## Conclusions

This study provides a valuable and complex set of data on genotoxic potential of the Sava River obtained from a single source which enables detection of the effects of genotoxic pollution on different levels. Result indicated differential sensitivity of applied bioassays in detection of genotoxic pressure. The standard and Fpg—modified comet assay showed higher potential in differentiation of the sites based on genotoxic potential in comparison with micronucleus assay and SOS/*umuC* test. Our data represent snapshot of the current status of the river which indicates the presence of genotoxic potential along the river which can be traced to the deterioration of quality of the Sava River by communal and industrial wastewaters.

## Supporting Information

S1 Fig Numbers of *E*. *coli* (MPN/100 mL) as an indication of faecal contamination Red lines represent borders between the classes of water quality [26]: I—slight, II–moderate, III—critical, IV–strong, V–excessive pollution. (TIF)

S1 Table Determination of soluble concentrations of elements in filtered (0.45 μm) water samples of the Sava River determined by ICP-MS. Measurement uncertainty better than ± 2%.*Hg concentration was determined from the whole water sample.
(DOCX)
