# Supplementary figures and images for: Evaluation of Genotoxic Pressure along the Sava River
Source: PLoS One. 2016 Sep 15;11(9):e0162450. doi: 10.1371/journal.pone.0162450 (PMC5025182; doi:10.1371/journal.pone.0162450)

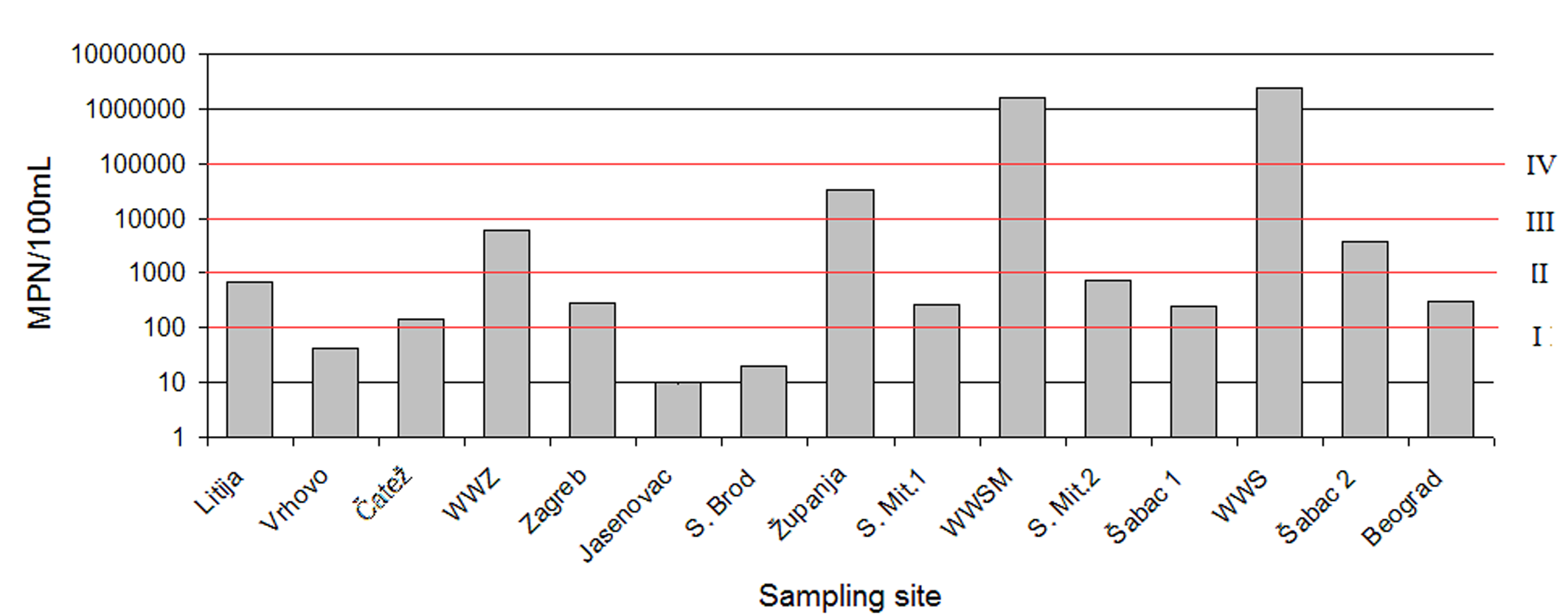

Supplement: S1 Fig — (TIF) [file pone.0162450.s001.tif]
