# Supplementary material for: Evaluation of Genotoxic Pressure along the Sava River
Source: PLoS One. 2016 Sep 15;11(9):e0162450. doi: 10.1371/journal.pone.0162450 (PMC5025182; doi:10.1371/journal.pone.0162450)
Supplement: S1 Table — Measurement uncertainty better than ± 2%.*Hg concentration was determined from the whole water sample. (DOCX) [file pone.0162450.s002.docx]

**S1 Table. Determination of soluble concentrations of elements in filtered (0.45 µm) water samples of the Sava River determined by ICP-MS. Measurement uncertainty better than ± 2%.**

| Sampling site | Cr (ng/mL) | Mn (ng/mL) | Fe (ng/mL) | Co (ng/mL) | Cd (ng/mL) | As (ng/mL) | Cu (ng/mL) | Ni (ng/mL) | Pb (ng/mL) | Zn (ng/mL) | Se (ng/mL) | Hg (ng/mL)* |
| --- | --- | --- | --- | --- | --- | --- | --- | --- | --- | --- | --- | --- |
| Vrhovo | 0.38 | 0.27 | 7.03 | 0.12 | 0.001 | 0.61 | 1.01 | 0.86 | 0.11 | 1.41 | 0.09 | 0.28 |
| Čatež | 0.29 | 0.39 | 1.40 | 0.08 | 0.001 | 0.57 | 0.90 | 0.47 | 0.06 | 0.83 | 0.07 | 0.46 |
| Zagreb | 0.27 | 0.34 | 2.93 | 0.12 | 0.005 | 0.69 | 1.21 | 0.51 | 0.11 | 1.02 | 0.07 | 0.29 |
| Jasenovac | 0.15 | 3.66 | 3.29 | 0.08 | 0.007 | 1.14 | 1.05 | 0.68 | 0.05 | 1.79 | 0.13 | 0.31 |
| Slavonski Brod | 0.22 | 0.39 | 16.9 | 0.11 | 0.001 | 1.25 | 1.11 | 5.73 | 0.04 | 0.71 | 0.07 | 0.22 |
| Županja | 0.23 | 0.43 | 29.4 | 0.08 | 0.001 | 1.49 | 1.12 | 2.80 | 0.04 | 0.33 | 0.11 | 0.13 |
| S. Mitrovica 2 | 0.28 | 0.26 | 1.59 | 0.04 | 0.001 | 1.51 | 0.90 | 1.38 | 0.02 | 0.60 | 0.08 | 0.34 |
| Šabac 1 | 0.31 | 1.54 | 2.57 | 0.04 | 0.001 | 1.56 | 0.85 | 1.15 | 0.04 | 0.21 | 0.08 | 0.25 |
| Belgrade | 0.28 | 7.42 | 27.2 | 0.05 | 0.015 | 1.91 | 1.68 | 1.33 | 0.05 | 2.18 | 0.10 | 0.27 |

*Hg concentration was determined from the whole water sample
